# The Association Between Estrogen Receptor-α and PIWIL3/piR-651/piR-823 Complex Regulates MI to MII Transposition in Normoresponder and Diminished Ovarian Reserve Cases

**DOI:** 10.3390/genes17020223

**Published:** 2026-02-11

**Authors:** Çağrı Öner, Damla Kolcuoğlu, Senem Aslan Öztürk, Nergis Özlem Kılıç, Duygu Kütük, Belgin Selam, İbrahim Orçun Olcay, Ertuğrul Çolak

**Affiliations:** 1 Department of Medical Biology, Medical Faculty, Kırklareli University, 39100 Kırklareli, Turkey; damlakolcuoglu@klu.edu.tr; 2 Department of Histology and Embryology, Medical Faculty, Maltepe University, 34844 Istanbul, Turkey; senemaslanozturk@gmail.com (S.A.Ö.); nergisozlemaltin@gmail.com (N.Ö.K.); duygukutukk@gmail.com (D.K.); 3 Department of Obstetrics and Gynecology, School of Medicine, Acibadem Mehmet Ali Aydinlar University, 34752 Istanbul, Turkey; 4 In Vitro Fertilization Laboratory, Bahçeci Umut Assisted Reproduction Center, 34662 Istanbul, Turkey; oolcay@bahceci.com; 5 Department of Biostatistics, Medical Faculty, Eskisehir Osmangazi University, 26040 Eskisehir, Turkey; ecolak@ogu.edu.tr

**Keywords:** estrogen receptor alpha, diminished ovarian reserve, normoresponder, PIWI-interacting RNA, PIWIL3

## Abstract

Background: Diminished ovarian reserve is characterized by a decrease in oocyte count and estrogen levels, which leads to infertility. The genetic and epigenetic mechanisms in MI to MII transition or complete MII phase in the oocyte maturation process estrogen receptor alpha and piRNA relationship were evaluated. Methods: This study analyzed 100 cumulus oophorous complex samples from normoresponder and DOR patients undergoing IVF, subdivided into metaphase I and metaphase II stages. To elucidate the ER-α, PIWIL3, piR-651, and piR-823 genes qRT-PCR was used and qualitative ER-α protein expressions were determined by immunohistochemistry. Pearson’s correlation analysis was utilized to evaluate the interactions between genes within each experimental group. Results: The DOR samples exhibited significant downregulation of ER-α gene and protein expression compared to the NOR controls. PIWIL3 gene, piR-651, and piR-823 expressions reduced in DOR MI and MII. Strong positive correlations among ER-α, PIWIL3, piR-651, and piR-823 were observed in NOR, whereas DOR showed weaker correlations and immunohistochemistry verified lower ER-α protein levels in DOR. Conclusions: The disruption of ER-α and piRNA-related gene networks in DOR may underlie the suboptimal maturation of oocytes, and monitoring ER-α, PIWIL3, piR-651, and piR-823 expressions could facilitate early determination of maturation stages and improve assessment of ovarian reserve. The potential for transposition to MII in NOR and DOR oocytes was observed in relation to the association between ER-α protein/gene expression and PIWIL3, which regulates ER-α. Moreover, piR-651 and piR-823, whose expressions depend on estrogen level, indirectly regulate oocyte maturation from MI to MII in both NOR and DOR epigenetically. We suggest that the MI and MII stages of oocytes could be determined earlier in NOR and DOR cases by controlling ER-α, PIWIL3, piR-651 and piR-823 expressions. These molecular markers indicate promise for diagnostic applications in reproductive medicine, warranting further validation in larger cohorts.

## 1. Introduction

Follicular aspiration fluid (FF) is a multifaceted, dynamic biological fluid that envelops the developing oocyte. The fluid that becomes wasted after oocytes are collected from patients applying to IVF centers contains cumulus oophorous complex (COC), which includes granulosa cells, the helper cells that nourish the oocyte [1]. Since the study of human oocytes in female infertility studies may cause ethical problems, COC cells in FF are a good source for genetic, epigenetic, and proteomic studies [2].

In oocyte maturation, there are two basic phases, called metaphase I (MI) and metaphase II (MII). After intracytoplasmic sperm injection (ICSI) of oocytes collected from patients applying to IVF centers, MI and MII terms were used to determine oocyte maturation. If the oocyte matures, it is called MII; if it does not mature and degenerates, it is called MI [3]. Various parameters are evaluated in the samples taken from the patients who apply to IVF centers and the case is diagnosed by taking these parameters into consideration [4]. If the patient’s oocyte amount is seven or more and does not have any genetic disease, it is classified as normoresponder (NOR); if the patient’s oocyte amount is six or less and biochemical or genetic problems are thought to be the cause of infertility, it is grouped as diminished ovarian reserve (DOR) [4]. Furthermore, DOR is defined by a drop in anti-Müllerian hormone (AMH), an elevation in baseline follicle-stimulating hormone (FSH > 10 IU/L on cycle days 2 to 3), or a reduction in the antral follicle count (AFC) (<5). While no substantial changes were seen in the clinical and hormonal profiles of DOR and NOR patients, levels of AMH, AFC, and oocyte counts were markedly decreased in DOR patients relative to NOR patients. Based on embryo development after treatment, they can be grouped into MI and MII.

The main biochemical parameters for female infertility or ovarian maturation are steroid hormones known as estrogens. These include estrone (E1), estradiol (E2), and estriol (E3). Among these, E2 is the most abundant and active estrogen, and it is widely accepted that estrogen exerts a critical influence on female reproduction via the two principal estrogen receptors (ERs), ER-α and ER-β [5] Estrogen receptors (ERs) are nuclear hormone receptors that mediate the effects of estrogens in various tissues, including the ovary. There are two main subtypes: ER-alpha (ER-α), encoded by the ESR1 gene; ER-beta (ER-β), encoded by the ESR2 gene. Both are expressed in ovarian tissue but have distinct roles in regulating ovarian function, folliculogenesis, and oocyte maturation [6]. ER-α is more involved in steroidogenesis and follicle growth, while the second principal subtype of estrogen receptor ER-β is crucial for granulosa cell function, folliculogenesis, and ovulation [7]. In animal models, knockout of ER-β leads to impaired follicle development, failure of oocyte maturation and ER-β regulates the expression of genes in granulosa cells that are essential for the response to the gonadotropins FSH and luteinizing hormone (LH), which are crucial for follicle development and ovulation [8]. Consequently, disruption of estrogen signaling, whether through the loss of estrogen receptors or aromatase, impedes the development of antral follicles [7]. While loss of ER-α results in infertility in both sexes, loss of ER-β specifically impairs female fertility due to defective follicle maturation and ovulation [9]. Therefore, both ER-α and ER-β regulate and compensate for each other but have unique functions in ovarian physiology as well.

Ovarian function depends on estrogen receptor-α (ER-α), which mediates estrogen signaling and influences follicle growth, maturation, and steroid hormone production [10,11,12]. Menstrual problems, symptoms of sex hormone insufficiency or fluctuation, and decreased fertility are among the clinical manifestations of DOR, which have a major impact on women’s quality of life and reproductive health [12]. In terms of DOR treatment approaches, hormone replacement therapy reduces the symptoms of estrogen insufficiency but has minimal impact on fertility and is unable to restore ovarian function [13]. Follicular atresia brought on by COC cell dysfunction results in a reduction in the quantity and quality of oocytes as well as estrogen levels, both of which are important components in the pathophysiology of DOR [14].

PIWI-interacting RNAs (piRNAs) are a novel class of small non-coding RNAs that were first determined in the pachytene stage of spermatogenesis [15]. The role of piRNAs during embryonic development is well known. In the biogenesis of piRNAs, at the first stage, the piRNA is cut from the region of origin. At the second stage, called the ping-pong cycle or amplification cycle, the piRNAs form complexes with PIWI proteins, become mature, and increase their number. They are then transferred to the nucleus, generally together with PIWIL3, and cause silencing of the relevant region by transposons [15].

piRNAs can trigger localized transposons and preserve reproductive integrity by safeguarding the germline genome from active mobile elements [16]. PIWI proteins and piRNAs have important roles in the development of competent oocytes in hamsters [17,18,19]. The existence of piRNAs has been identified in the germline of female mammalian species, as well as a class of female-specific PIWIL3-piRNA complexes in embryonic gonads [20,21]. Moreover, the four distinct PIWI proteins (PIWIL1–PIWIL4) that are found in most mammalian genomes are what piRNAs interact with. Human, macaque, bovine, and hamster oocytes have significant and specific expressions of the PIWIL3 protein and/or Piwil3 gene, which is not present in the genomes of mice or rats [17,18,19,22,23].

piR-651 inhibits cell death while promoting cell invasion, migration, and proliferation [24]. By inhibiting the apoptotic caspase-3/Bax signaling pathway, piR-823 promotes endothelial cell growth [25]. Another study found that piR-823 reduced cell proliferation by inducing DNA methylation via DNA methyltransferases 3A and 3B (DNMT3A and DNMT3B) [26].

In this study, we aimed to determine the genetic and protein expression of the ER-α and piRNA complexes (PIWIL3/piR-651/piR-823) in COC cells from MI and MII NOR and DOR cases using waste FF.

## 2. Materials and Methods

### 2.1. Sample Selection

The study participants comprised women who were referred to the Bahçeci Health Group Umut IVF Center between 22 April 2024 and 22 August 2025 (Ethics Committee No.: 2024/06-08, Date: 14 March 2024 and 2025/19-11, Date: 2 October 2025; all study participants provided written informed consent). The COC cells were aspirated directly from follicles and COC samples were examined from the follicular aspiration fluids of NOR MI, NOR MII, DOR MI, and DOR MII patients in this research. A total of 100 samples were studied in this research. Each of the NOR and DOR groups had 50 COC samples taken: NOR samples (*n* = 50) were also subgrouped as NOR MI (*n* = 25) and NOR MII (*n* = 25). Furthermore, DOR samples (*n* = 50) were subgrouped as DOR MI (*n* = 25) and DOR MII (*n* = 25). The inclusion criteria for the NOR (control) group included cases of AFC 7–12 and anti-Müllerian hormone (AMH) levels of 1.0–3.5 ng/mL. The selection criteria further stipulated the presence of both ovaries and their visibility on ultrasound, with exclusion from any prior history of ovarian surgery. The following requirements must be fulfilled by DOR cases: AMH values <1 ng/mL, AFC <5–7. As COC cells were to be aspirated and gathered based on MI and MII characteristics in both the NOR and DOR groups, patients were required to supply a minimum of two oocytes per group. This research did not include cases from either group in which just one oocyte was obtained. Endometriosis that was diagnosed by laparoscopy or imaging, Polycystic Ovary Syndrome (PCOS), thyroid dysfunction (abnormal TSH), history of previous ovarian surgery, and known genetic disorders affecting fertility, such as Turner syndrome and Fragile X premutation, were the exclusion criteria of this study. The samples collected from each group were stored in a −80 °C freezer until they were analyzed.

### 2.2. Isolation of Cumulus Oophorous Complex (COC) Cells from Follicular Fluid

Oocyte pickup (OPU) was used to isolate COC cells from the follicular fluid (FF). Every individual’s granulosa cell surrounding the antrum near the corona radiata was eliminated by the denuding process. Stereomicroscopy was used to manually separate COC cells from oocytes using sensitive glass pipettes. COC cells were gently pipetted up and down in TCM199 media containing 80 IU of hyaluronidase/mL and 20% fetal calf serum (FCS). Using a stereomicroscope (Nikon SMZ-1500, Nikon, Berlin, Germany), polar body (PB) visualization was performed to examine the meiotic stage of the oocytes after all COC cells had been completely removed. In accordance with MI and MII oocytes, the COC cells from the collected cases were put in centrifuge tubes in their FF individually. After that, FF containing COC cells was centrifuged for 20 min at +4 °C and 2800 rpm. After the supernatant was discarded, pellets containing COC cells were centrifuged in phosphate-buffered saline (PBS) for five minutes at room temperature and 1850 rpm.

### 2.3. Total RNA Isolation and Reverse Transcription to cDNA

Reverse transcription of total RNAs produced cDNA, and total RNA isolation was carried out in accordance with the NGE024 NucleoGene QuickEX Total RNA Isolation Kit procedure (Nucleogene, Istanbul, Turkey). At 25 °C for five minutes, 50 °C for thirty minutes, and 85 °C for five minutes, the cDNA reverse transcription was carried out according to the manufacturer’s protocol (NGMM020, Nucleogene 5X cDNA synthesis kit, Nucleogene, İstanbul, Turkey).

### 2.4. Quantitative Real-Time Polymerase Chain Reaction (qRT-PCR)

Estrogen Receptor-Alpha (ER-α), PIWIL3, piR-651, piR-823, and Glyseralde-hide-3-phosphate dehydrogenase (GAPDH) were all amplified using SYBR Green primer sets that were created and provided by Bmlabosis (Ankara, Turkey). The cDNA samples were kept at −80 °C (Haier Biomedical, Qingdao, China) for additional processing after being tested for RNA using a Microplate Reader (Spectrostar Nano, BMG-Labtech, Ortenberg, Germany). Table 1 lists the primer sequences that were used in the qRT-PCR method. ΔCT values (Livak & Schmittgen, 2001 [27]) were calculated using GAPDH as an internal reference (Table 1). The qRT-PCR conditions were set as: 95 °C for 15 s, 60 °C for 30 s, and 40 cycles of 95 °C for 30 s.

### 2.5. Immunohistochemical Staining

The Envision IHC Staining kit (Dako Omnis, Agilent, Singapore) was used to examine ER-α protein expression. On a polylysine-coated slide (Objekttrager, Adhesive-Silane, Heidelberg, Germany), the COC cells of the NOR and DOR samples were collected by smears and allowed to dry at room temperature. ER-α primary antibody (E-AB-31378, Elabscience) was fixed for 15 min in 10% formaldehyde solution, then diluted in 0.1% (*w*/*v*) bovine serum albumin (BSA) and incubated with primary antibodies at +4 °C for the whole night. To prevent non-specific binding, the samples were treated with EnVision FLEX Peroxidase-Blocking Reagent (Dako Omnis, Singapore) for three minutes at room temperature the next day. They were then rinsed with PBS (Sigma Aldrich, Taufkirchen, Germany). EnVision Flex HRP (Dako Omnis, Singapore) was applied to the samples as a secondary antibody and allowed to incubate at room temperature for 20 min. The slides were washed with PBS twice after 20 min. For immunolocalization, the samples were mixed with EnVision Flex Substrate Working solution (DAB/Chromogen+Substrate Buffer), allowed to incubate at room temperature for five minutes, and then rinsed with PBS again. Samples were then rinsed with deionized water and PBS after being treated with hematoxylin stain for 15 min. Following a 1.5 min incubation period with eosin dye, samples were rinsed with PBS, and the preparation was coated with glycerol. Using 20× and 40× microscope magnifications (Olympus, Hamburg, Germany), eight randomly selected fields were taken. Both quantitative and qualitative data were obtained by counting, dividing, and statistically analyzing the stained and unstained cells.

### 2.6. Retrospective Data of ER-α

Retrospective data were gathered on patients admitted to Istanbul Bahçeci Umut IVF Center between 22 April 2024 and 22 August 2025. The quantification of ER-α levels was performed according to the protocol provided by the Human Estrogen Receptor Alpha (ER-α) ELISA Kit (Catalogue No. ab108918, Abcam, Cambridge, UK). In summary, samples and standards were added to the wells, incubated, washed, and developed in accordance with the kit protocol. Absorbance was measured at 450 nm, and concentrations were calculated from a standard curve. The biochemical examination of ER-α was included in these retrospective data for females.

### 2.7. Statistical Evaluation

For all statistical analyses, the IBM SPSS Statistics 26.0 software was used. Continuous variables were given a normal distribution by applying the Kolmogorov–Smirnov compatibility test. The quantitative non-normally advanced distribution, independent samples of the Kruskal–Wallis test, and Bonferroni’s correction test were compared. Pearson’s correlation test was used to assess comparisons between groups. The ER-α protein expression values were evaluated using ANOVA and the Tukey post hoc test. The gathered data are presented in the text as mean ± standard deviation (SD). Group-to-group significance criteria (*p* < 0.05; *p* < 0.01; and *p* < 0.001 or *p* > 0.05) were established.

## 3. Results

There were no statistically significant differences in BMI among the four groups, supporting the comparability of the groups and the validity of this study’s conclusions (*p* > 0.05). There were significant changes in the age of the cases between NOR MI, DOR MI, and DOR MII (*p* < 0.05); and between NOR MII, DOR MI and DOR MII (*p* < 0.05). Furthermore, there was a significant change in oocyte count between NOR MI and NOR MII (*p* < 0.0001); between NOR MII, DOR MI, and DOR MII (*p* < 0.0001); and between DOR MI and DOR MII (*p* < 0.05). Moreover, the fertilization rates of the cases were statistically different between NOR MI and NOR MII (*p* < 0.0001); and between NOR MII, DOR MI, and DOR MII (*p* < 0.0001; Supplementary Table S3).

### 3.1. Gene Expression Analysis

**Figure 1:**
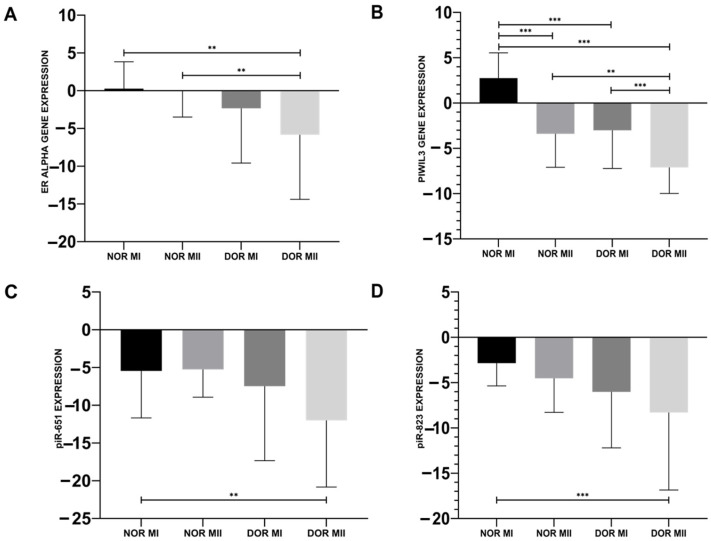
Gene expression results for the differences between NOR MI (*n* = 25), NOR MII (*n* = 25), DOR MI (*n* = 25), and DOR MII (*n* = 25). (**A**) ER-α gene expressions; (**B**) PIWIL3 gene expressions; (**C**) piR-651 expressions; (**D**) piR-823 expressions in NOR MI, NOR MII, DOR MI, and DOR MII groups (** *p* < 0.05; *** *p* < 0.001).

The ER-α gene expression of DOR MII (−5.839 ± 8.55) was downregulated compared to both the NOR MI (0.275 ± 3.559) and NOR MII (−0.069 ± 3.425) groups (Figure 1A; *p* < 0.05). ER-α gene expression in DOR MI (−2.328 ± 7.258) was downregulated compared to NOR MI, but this downregulation was not statistically significant (Figure 1A; *p* > 0.05).

The decrease in PIWIL3 gene expression was observed in DOR MII (−7.103 ± 2.878) compared to the NOR MI (2.745 ± 2.792; *p* < 0.001), NOR MII (−3.4 ± 3.693; *p* < 0.05), and DOR MI (−3.01 ± 4.214; *p* < 0.001) groups (Figure 1B). The induced PIWIL3 gene expression of NOR MII and DOR MI was detected compared to NOR MI (Figure 1B; *p* < 0.001).

piR-651 expression reduced in DOR MII (−11.997 ± 8.835) compared to the NOR MI (−5.450 ± 6.231), and was statistically significant (Figure 1C; *p* < 0.05). However, there is also an increase in piR-651 expression in the NOR MII (−5.251 ± 3.68) and DOR MI (−7.473 ± 9.864) groups; this induction was not statistically significant (Figure 1C; *p* > 0.05). A statistically significant decrease in piR-823 was observed in DOR MII (−8.302 ± 8.552) compared to NOR MI (−2.849 ± 2.513; *p* < 0.001), although the decrease in the NOR MII (−4.52 ± 3.768) and DOR MI (−6.014 ± 6.186) groups was not statistically significant (Figure 1D; *p* > 0.05).

### 3.2. Immunohistochemical Protein Expression Analysis

**Figure 2:**
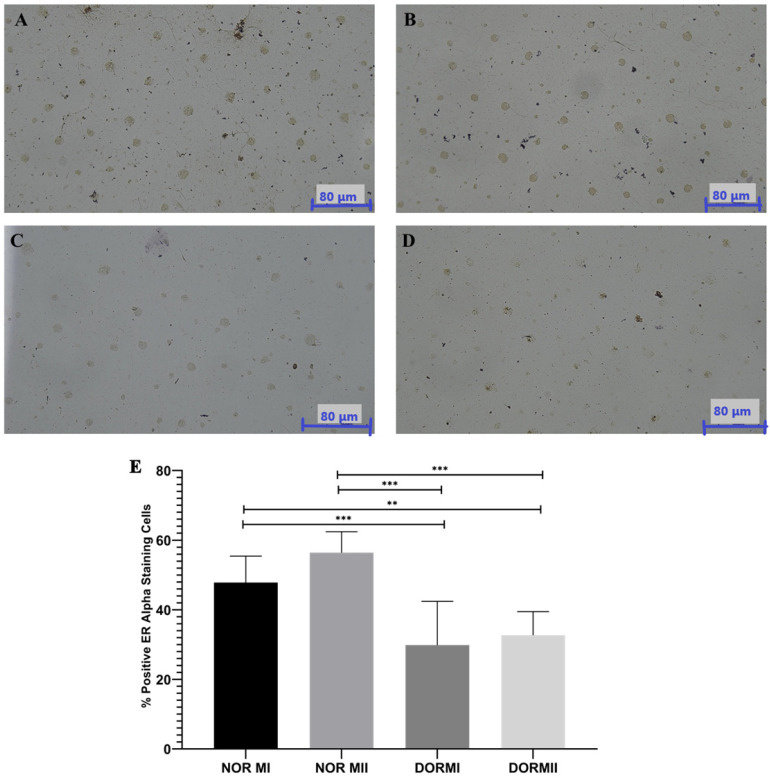
The immunohistochemistry staining photographs with ER-α antibody in (**A**) NOR MI (*n* = 25); (**B**) NOR MII (*n* = 25); (**C**) DOR MI (*n* = 25); (**D**) DOR MII (*n* = 25; Magnification Ratio: 40×). The number of the ER-α positive cells that occurred in the NOR MI and NOR MII groups (Figure 2A,B) is higher than in the DOR MI and DOR MII groups (Figure 2C,D). (**E**) The graph indicates the % of positive ER-α-stained cells by immunohistochemistry. (** *p* < 0.05; *** *p* < 0.001; *p* > 0.05).

The immunohistochemistry findings for ER-α indicated that the control group (NOR MI and NOR MII) had significantly elevated protein expression levels relative to the experimental groups (DOR MI and DOR MII; *p* < 0.001; Figure 2). The ER-α protein expression was 47.86 ± 7.60 for NOR MI and 56.43 ± 5.97 for NOR MII (Figure 2A,B), but DOR MI (29.86 ± 12.59) and DOR MII (32.71 ± 6.78) exhibited lower ER-α protein expression, respectively (*p* < 0.001; Figure 2C,D). ANOVA statistical analysis indicated a significant difference between groups (*p* < 0.001), and post hoc Tukey testing verified that both the DOR MI and DOR MII groups exhibited significantly decreased ER-α expression relative to the control groups (*p* < 0.05; Figure 2). There was a statistically significant decrease between NOR MI and DOR MI (*p* < 0.001); NOR MI and DOR MII (*p* < 0.05); NOR MII and DOR MI (*p* < 0.001); and NOR MII and DOR MII (*p* < 0.001; Figure 2). There is no significant difference between NOR MI and NOR MII (*p* > 0.05), nor between DOR MI and DOR MII (*p* > 0.05; Figure 2).

### 3.3. Retrospective Evaluation of ER-α

**Figure 3:**
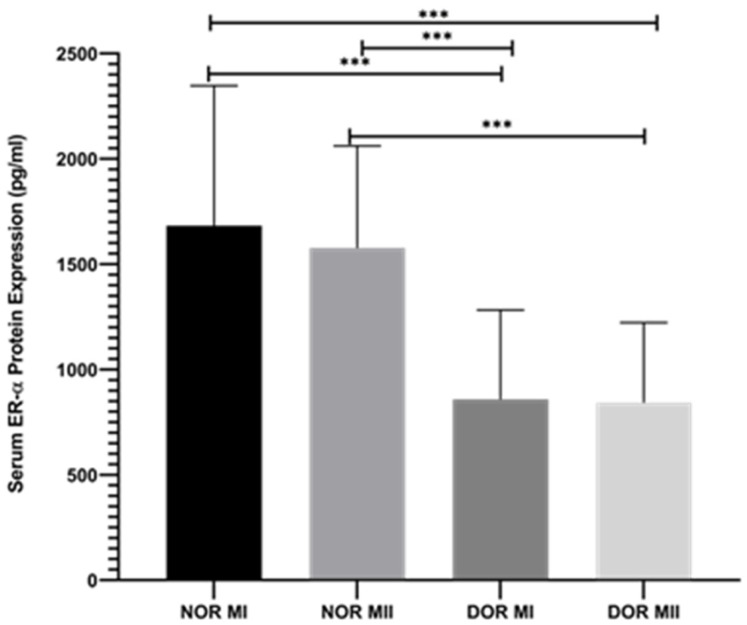
The protein expression retrospective evaluation of ER-α between NOR MI (*n* = 25), NOR MII (*n* = 25), DOR MI (*n* = 25), and DOR MII (*n* = 25; *** *p* < 0.001).

According to the retrospective data obtained from the biochemistry laboratory, ER-α protein ex-pression was decreased in the NOR MI (1682.8 ± 663.92 pg/mL) and DOR MI (858.532 ± 424.087 pg/mL) groups (Figure 3; *p* < 0.001). Furthermore, this decrease was observed between the NOR MII (1577.04 ± 484.436 pg/mL) and DOR MII (842.932 ± 379.185 pg/mL) groups (Figure 3; *p* < 0.001). Moreover, ER-α protein expressions are downregulated between NOR MI and DOR MII (Figure 3; *p* < 0.001). A similar decrease was observed between NOR MII and DOR MI (Figure 3; *p* < 0.001).

### 3.4. Results of Pearson’s Correlation Test

**Figure 4:**
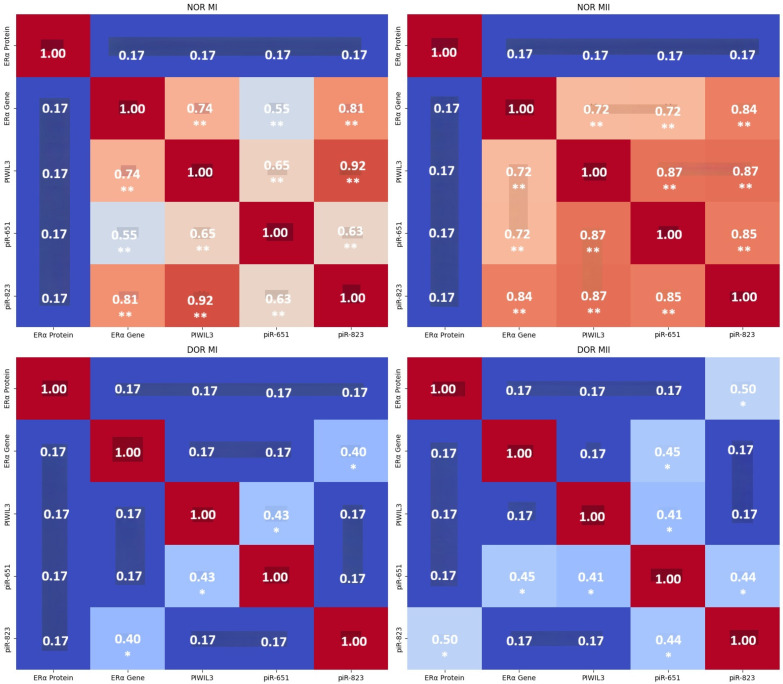
Combined comparative heatmap with NOR MI (*n* = 25), NOR MII (*n* = 25), DOR MI (*n* = 25), and DOR MII (*n* = 25) and statistical significance markers. Each subplot represents one group for direct comparison. Darker red squares indicate stronger positive correlation; blue squares indicate weaker or negative correlation. NOR MI and NOR MII: Dense clusters of ** markers among the ER-α, PIWIL3, piR-651, and piR-823 genes show strong, highly significant correlations. DOR MI: Sparse * markers indicate only a few moderate correlations remain. DOR MII: Slight improvement with ER-α protein and piR-823; piR-651 and others show significance (Pearson’s correlation test; * *p* < 0.05; ** *p* < 0.01, Supplementary Material 1). The combined comparative heatmap was generated by Microsoft 365 Copilot, GPT-5–based.

In the NOR MI group, ER-α protein expression showed no significant correlations with other parameters (ER-α, PIWIL3, piR-651, and piR-823; *p* > 0.05). ER-α gene expression showed strong, significant positive correlations with PIWIL3 (r = 0.742), piR-651 (r = 0.550), and piR-823 (r = 0.811, *p* < 0.01). PIWIL3 gene expression showed strong, significant positive correlations with ER-α, piR-651 (r = 0.650), and piR-823 (r = 0.923, *p* < 0.01). piR-651 expression had significant positive correlations with ER-α, PIWIL3, and piR-823 (r = 0.634, *p* < 0.01; (Figure 4) and (Supplementary Material 1).

In NOR MII, ER-α protein showed no significant correlations with other parameters (*p* > 0.05). The ER-α gene showed significant positive correlations with PIWIL3 (r = 0.718), piR-651 (r = 0.725), and piR-823 (r = 0.843, *p* < 0.01). The PIWIL3 gene indicated significant positive correlations with ER-α, piR-651 (r = 0.866), and piR-823 (r = 0.872, *p* < 0.01). piR-651 had significant positive correlations with ER-α, PIWIL3, and piR-823 (r = 0.850, *p* < 0.01; (Figure 4) and (Supplementary Material 1).

In the DOR MI group, ER-α protein expression data showed no significant correlations with other parameters (*p* > 0.05). The ER-α gene showed a significant positive correlation with piR-823 (r = 0.404). Furthermore, the PIWIL3 gene had a significant positive correlation with piR-651 (r = 0.432, *p* < 0.05; (Figure 4) and (Supplementary Material 1).

Opposite to the other groups, ER-α protein showed a significant positive correlation with piR-823 (r = 0.496) in the DOR MII group. The ER-α gene indicated a significant positive correlation with piR-651 (r = 0.452). Furthermore, the PIWIL3 gene had a significant positive correlation with piR-651 (r = 0.414). Moreover, piR-651 had significant positive correlations with ER-α, PIWIL3, and piR-823 (r = 0.439, *p* < 0.05; (Figure 4) and (Supplementary Material 1).

## 4. Discussion

Reduced ovarian reserve is characterized by a decrease in both the number and quality of oocytes and supporting cells. This process disrupts the transcriptional landscape of ovarian cells, including ER-α-regulated genes, and impairs normal follicle development and ovulation [28]. Recent single-cell sequencing studies in animal models show that ER-α deletion or downregulation significantly alters the expression of genes involved in ovarian function, infertility, and steroidogenesis. This highlights the central role of ER-α in maintaining ovarian health and function [29].

While this study provides valuable insights into the molecular mechanisms underlying oocyte maturation in NOR and DOR cases, its limitations include a small sample size, a narrow molecular focus, and the use of proxy cells. Future studies should address these limitations to confirm and extend the findings. This study included 100 samples divided into NOR and DOR groups, with further subdivision into MI and MII stages (25 samples per subgroup). While this is a reasonable sample size for a pilot or exploratory study, it may not be sufficient to capture the full biological variability, especially for complex genetic and epigenetic interactions. Larger cohorts would strengthen statistical power and generalizability. Furthermore, genetic and protein analyses were performed on cumulus oophorous complex (COC) cells from follicular fluid, not directly on oocytes. While COC cells are a good proxy, they may not fully reflect the molecular state of the oocyte itself. Moreover, this study relies on gene and protein expression data and correlation analyses. Functional experiments (e.g., knockdown or overexpression studies, mechanistic assays) would be needed to confirm causality and clarify the biological roles of these molecules.

Epigenetic modifications (such as DNA methylation, histone modifications, and non-coding RNAs) play a crucial role in regulating gene expression in ovarian granulosa cells and oocytes. In DOR, these modifications can silence or reduce gene expression, such as ER-α, affecting hormone production and follicle development. Non-coding RNAs (miRNAs, lncRNAs, and piRNAs) also contribute to this regulation, impacting granulosa cell function and oocyte maturation [30]. PIWIL3 is a member of the PIWI protein family, which binds to piRNAs [31,32]. These complexes are crucial for gene silencing, transposon repression, and epigenetic regulation in germ cells [33]. In oocytes, PIWIL3 forms complexes with specific piRNAs, notably piR-651 and piR-823. These piRNAs regulate cell survival, proliferation, and epigenetic modifications, such as DNA methylation [34,35,36,37]. The PIWIL3/piRNA complex influences the expression of estrogen receptor alpha (ER-α) at both gene and protein levels. In normoresponder (NOR) oocytes, there is a strong positive correlation between PIWIL3 and ER-α gene expression, indicating coordinated regulation. This regulation is likely mediated through epigenetic mechanisms, in which piRNAs guide PIWIL3 to specific genomic regions to modulate ER-α transcription, possibly by altering chromatin structure or DNA methylation status.

ER-α gene and protein expression levels are significantly higher in NOR cases and in DOR cases at both the MI and MII stages. This suggests that ER-α is associated with healthy oocyte maturation and progression from MI to MII. Furthermore, ER-α interacts with PIWIL3 and piRNAs (piR-651 and piR-823), forming a regulatory network that influences oocyte maturation. In NOR cases, strong positive correlations among the ER-α, PIWIL3, piR-651, and piR-823 genes indicate coordinated regulation, which is essential for normal oocyte development. In DOR cases, these interactions are disrupted, leading to impaired oocyte maturation. The decrease in ER-α expression is linked to poor progression from MI to MII, which is a hallmark of DOR.

Estrogen levels, sensed and mediated by ER-α, influence the expression of piR-651 and piR-823. This creates a feedback loop in which ER-α promotes the expression of these piRNAs, which, in turn, through PIWIL3, regulate ER-α expression and activity. This feedback ensures that oocyte maturation (maturation from MI to MII) is tightly controlled by both hormonal (estrogen) and epigenetic (piRNA/PIWIL3) signals. In DOR cases, PIWIL3, piR-651, piR-823, and ER-α are downregulated. The correlations between these molecules are weaker, indicating a breakdown in the regulatory network. This disruption leads to impaired oocyte maturation and poor progression from MI to MII. The PIWIL3/piRNA complex may induce DNA methylation and other epigenetic changes that silence or activate genes involved in oocyte maturation. For example, piR-823 has been shown to regulate DNA methyltransferases, affecting cell proliferation and differentiation (Supplementary Table S2).

Estrogen receptor alpha (ER-α) is essential for oocyte maturation, namely in the progression from metaphase I (MI) to metaphase II (MII) during ovarian follicle development. DOR is associated with a reduced number and reduced quality of ovarian follicles, leading to altered local hormone levels (such as lower estrogen levels). Since ER-α level is partly regulated by estrogen, a decrease in estrogen can downregulate both ER-α protein and gene expressions in ovarian tissue. Additionally, increased follicular atresia and cellular stress in DOR can further suppress ER-α protein and gene expressions [33]. Studies in both humans and animal models confirm that DOR is linked to lower ER-α immunoreactivity in ovarian tissue. This is reflected in reduced ER-α immunohistochemical staining, as seen in our results. The changes are multifactorial, involving hormonal, epigenetic, and cellular stress pathways [28,29,30,38]. Both DOR MI and DOR MII have significantly lower ER-α protein expression compared to NOR MI and NOR MII. The most pronounced differences are between the NOR (control) groups and the DOR (experimental) groups, indicating a strong effect of the experimental condition on ER-α expression. The impact of diminished ovarian reserve on ovarian tissue health, hormone signaling, and cellular function is reflected in the lower ER-α protein expressions in DOR groups relative to NOR groups. This is in line with research showing that decreased reserve and ovarian aging are associated with altered ovarian responsiveness and reduced expression of estrogen receptors.

In the NOR MI and NOR MII groups, strong positive correlations among the ER-α, PIWIL3, piR-651, and piR-823 genes were detected. Furthermore, ER-α protein shows no significant correlation with other parameters. These results suggest a tightly linked regulatory network between the ER-α gene and piRNA-related genes in NOR MI and NOR MII. For DOR MI, there were weak overall correlations. The correlations between the ER-α gene and piR-823, and between PIWIL3 and piR-651 are significant in NOR MI. This result indicates disrupted gene-piRNA interactions in diminished ovarian reserve (MI stage). For DOR MII, we observed moderate correlations between ER-α protein and piR-823, between the ER-α gene and piR-651, and between piR-651 and piR-823. These data for DOR MII suggest partial restoration or alternative regulatory pathways at the MII stage. In the NOR MI and NOR MII groups, strong gene-piRNA connectivity implies coordinated regulation during normal ovarian function. On the other hand, in the DOR groups, the correlation strength decreases, indicating impaired molecular interactions. ER-α protein becomes more relevant in DOR MII, possibly compensating for gene-level disruptions.

**Figure 5:**
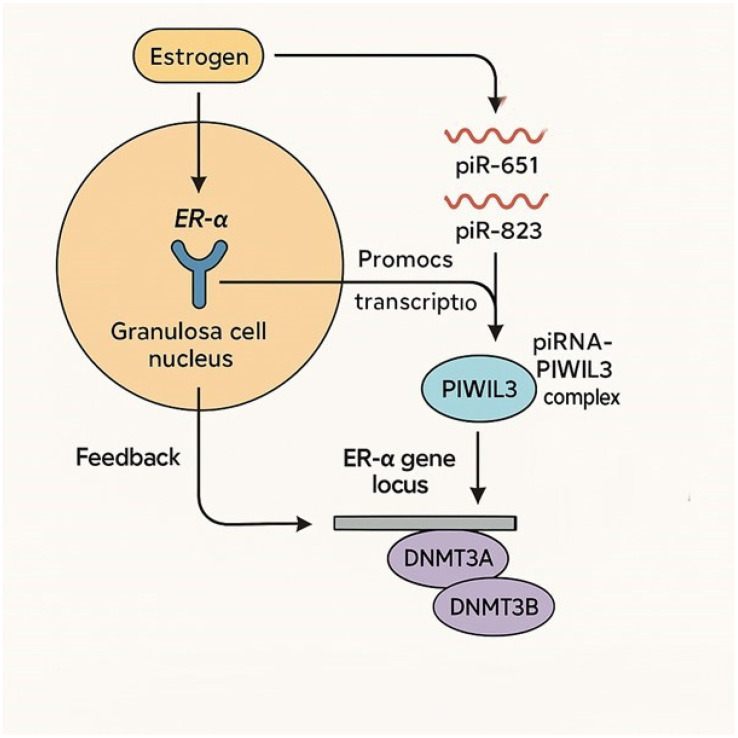
The possible regulation pathway of PIWIL3/piR-651/piR-823 and ER-α. Estrogen binds to ER-α in the nucleus of granulosa or cumulus cells. ER-α acts as a transcription factor, promoting the expression of specific piRNAs (such as piR-651 and piR-823). piRNAs bind to the PIWIL3 protein, forming a piRNA–PIWIL3 complex in the cytoplasm. This complex translocates to the nucleus and targets the ER-α gene locus. The complex recruits DNA methyltransferases (DNMT3A, DNMT3B), which modify the chromatin structure at the ER-α promoter, regulating its transcription (either activating or repressing, depending on context). Feedback loop: ER-α expression influences piRNA levels, ensuring tight control over oocyte maturation (feedback loop).

According to our obtained data, piRNAs (piR-651 and piR-823) could regulate ER-α expression through epigenetic mechanisms and by forming complexes with PIWI proteins, particularly PIWIL3 (Figure 5).

## 5. Conclusions

This study suggests that monitoring ER-α, PIWIL3, piR-651, and piR-823 expressions could help determine the MI and MII stages of oocytes earlier in both NOR and DOR cases, potentially aiding in the diagnosis and management of ovarian reserve status. Furthermore, this research identifies a disrupted regulatory network involving ER-α and piRNA-related genes in DOR cases, which may underlie poor oocyte maturation. The reduction in ER alpha expression in DOR is driven by a combination of epigenetic silencing, hormonal changes due to follicle depletion, cellular aging, and altered gene regulation in ovarian tissue. These mechanisms collectively impair ovarian function and responsiveness, distinguishing DOR from NOR cases. These results highlight the mechanism between ER-α and PIWIL3/piR-651/piR-823 cascade in NOR and DOR cases. Furthermore, the results indicate the potential of these molecular markers (PIWIL3, piR-651, and piR-823) for early diagnosis of NOR MI, DOR MI, NOR MII, and DOR MII. Moreover, these findings suggest preliminary data for further research is needed to confirm these findings and explore therapeutic implications.

## Figures and Tables

**Table 1:** The primer sequences that were used in the qRT-PCR method.

Accession Number	Gene/piRNA	Forward Primer	Reverse Primer	Melting Temperature (Tm)
**NM-001122741**	**ER-α**	5′-GCACCCTGAAGTCTCTGGAA-3′	5′-TGGCTAAAGTGGTGCATGAT-3′	58 °C
**NM_001008496.3**	**PIWIL3**	5′-TTTCCAAAGAACTCACGCCC-3′	5′-ACATCGGCACAGAGGGTAAT-3′	57 °C
**DQ570563.1**	**piR-651**	5′-AGAGAGGGGCCCCCCGTGCCTTG-3′	5′-CTTCTTATGGAGCCTGGGACTCTGACC-3′	66 °C
**DQ570735.1**	**piR-823**	5′-AGCGTTTTGGTGGTATAGTGGT-3′		57 °C
**NG-007073.2**	**GAPDH**	5′-CGAGGGGGGAGCCAAAAGGG-3′	5′-TGCCAGCCCCAGCGTCAAAG-3′	55 °C

## Data Availability

The original contributions presented in this study are included in the article/Supplementary Materials. Further inquiries can be directed to the corresponding author.

## Supplementary Materials

The following supporting information can be downloaded at: https://www.mdpi.com/article/10.3390/genes17020223/s1, Table S1: Table of the correlations between ER-α, PIWIL3 gene expressions, piR-651, piR-823 expression, and ER-α protein expression; Table S2: PIWIL3–piRNA regulation of ER-α; Table S3: Baseline demographic and clinical characteristics of the study groups.

## Abbreviations

The following abbreviations are used in this manuscript:

NOR	Normoresponder
DOR	Diminished Ovarian Reserve
FF	Follicular Fluid
AMH	Anti-Müllerian Hormone
FSH	Follicle-Stimulating Hormone
AFC	Antral Follicle Count
LH	Luteinizing Hormone
COC	Cumulus Oophorous Complex
OPU	Oocyte Pickup
piRNAs	PIWI-Interacting RNAs
ICSI	Intracytoplasmic Sperm Injection
E1	Estrone
E2	Estradiol
E3	Estriol
ERα	Estrogen Receptor Alpha
ERβ	Estrogen Receptor Beta
