# Supplementary material for: The Association Between Estrogen Receptor-α and PIWIL3/piR-651/piR-823 Complex Regulates MI to MII Transposition in Normoresponder and Diminished Ovarian Reserve Cases
Source: Genes (Basel). 2026 Feb 11;17(2):223. doi: 10.3390/genes17020223 (PMC12940606; doi:10.3390/genes17020223)
Supplement: Supplementary file 1 [file genes-17-00223-s001.zip › genes-4140375-supplementary.pdf]

Supplementary Materials (Table S1 and Table S2)

Table S1. Correlations between ER alpha, PIWIL3 gene expressions, piR-651, piR-823 expression, and ER alpha protein expression

			**ER-α Protein**	**ER-α**	**PIWIL3**	**piR-651**	**piR-823**
**NOR MI**	**ER-α Protein**	Pearson Correlation	1	,277	,008	,052	,075
		Sig. (2-tailed)	-	,180	,968	,804	,720
		N	25	25	25	25	25
	**ER-α**	Pearson Correlation	,277	1,000	,742**	,550**	,811**
		Sig. (2-tailed)	,180	-	,000	,004	,000
		N	25	25	25	25	25
	**PIWIL3**	Pearson Correlation	,008	,742**	1,000	,650**	,923**
		Sig. (2-tailed)	,968	,000	-	,000	,000
		N	25	25	25	25	25
	**piR-651**	Pearson Correlation	,052	,550**	,650**	1,000	,634**
		Sig. (2-tailed)	,804	,004	,000	-	,001
		N	25	25	25	25	25
	**piR-823**	Pearson Correlation	,075	,811**	,923**	,634**	1,000
		Sig. (2-tailed)	,720	,000	,000	,001	-
		N	25	25	25	25	25
**NOR MII**	**ER-α Protein**	Pearson Correlation	1,000	-,149	-,153	-,083	-,096
		Sig. (2-tailed)	-	,476	,465	,693	,648
		N	25	25	25	25	25
	**ER-α**	Pearson Correlation	-,149	1,000	,718**	,725**	,843**
		Sig. (2-tailed)	,045	-	,000	,000	,000
		N	25	25	25	25	25
	**PIWIL3**	Pearson Correlation	-,153	,718**	1,000	,866**	,872**
		Sig. (2-tailed)	,465	,000	-	,000	,000
		N	25	25	25	25	25
	**piR-651**	Pearson Correlation	-,083	,725**	,866**	1,000	,850**
		Sig. (2-tailed)	,693	,000	,000	-	,000
		N	25	25	25	25	25
	**piR-823**	Pearson Correlation	-,096	,843**	,872**	,850**	1,000
		Sig. (2-tailed)	,648	,000	,000	,000	-
		N	25	25	25	25	25
**DOR MI**	**ER-α Protein**	Pearson Correlation	1,000	,101	,182	-,133	,149
		Sig. (2-tailed)	-	632	,385	,526	,476
		N	25	25	25	25	25
	**ER-α**	Pearson Correlation	,101	1,000	,277	,380	,404*
		Sig. (2-tailed)	,632	-	,180	,061	,045
		N	25	25	25	25	25
	**PIWIL3**	Pearson Correlation	,182	,277	1,000	,432*	,315
		Sig. (2-tailed)	,385	,180	-	,031	,125
		N	25	25	25	25	25
	**piR-651**	Pearson Correlation	-,133	,380	,432*	1,000	,351
		Sig. (2-tailed)	,526	,061	,031	-	,085
		N	25	25	25	25	25
	**piR-823**	Pearson Correlation	,149	,404*	,315	,351	1,000
		Sig. (2-tailed)	,476	,045	,125	,085	-
		N	25	25	25	25	25
**DOR MII**	**ER-α Protein**	Pearson Correlation	1,000	-,072	,148	,165	,496*
		Sig. (2-tailed)	-	,733	,481	,432	,012
		N	25	25	25	25	25
	**ER-α**	Pearson Correlation	-,072	1,000	,101	,452*	,112
		Sig. (2-tailed)	,733	-	,629	,023	,594
		N	25	25	25	25	25
	**PIWIL3**	Pearson Correlation	,148	,101	1,000	,414*	,234
		Sig. (2-tailed)	,481	,629	-	,039	,261
		N	25	25	25	25	25
	**piR-651**	Pearson Correlation	,165	,452*	,414*	1,000	,439*
		Sig. (2-tailed)	,432	,023	,039	-	,028
		N	25	25	25	25	25
	**piR-823**	Pearson Correlation	,496*	,112	,234	,439*	1,000
		Sig. (2-tailed)	,012	594	,261	,028	-
		N	25	25	25	25	25

***Correlation is significant at the 0.05 level (2-tailed).**
****Correlation is significant at the 0.01 level (2-tailed).**

Table S2: PIWIL3–piRNA Regulation of ER-α

Step	Molecule(s) Involved	Mechanism	Outcome
1	piR-651, piR-823, PIWIL3	Complex formation (gene silencing)	Targets ER-α gene (Regulates target gene expression)
2	PIWIL3/piRNA complex	Recruits DNMTs, modifies chromatin (Epigenetic modulation)	Alters ER-α transcription (Controls ER-α gene/protein levels)
3	ER-α	Regulates piRNA expression	Feedback control (Estrogen-dependent feedback)
4	ER-α, PIWIL3, piR-651, piR-823	Coordinated regulation	Ensures proper MI to MII transition<br>Proper oocyte maturation

Table S3. Baseline demographic and clinical characteristics of the study groups.

Parameters	NOR MI (n=25)	NOR MII (n=25)	DOR MI (n=25)	DOR MII (n=25)	p-value
**Age (years)**	33,3	33,3	38,4	38,4	p<0.05
**BMI (kg/m²)**	26,1	26,1	25,3	25,3	p>0.05
**Oocyte Count**	2,04	7,32	1,08	2,56	p<0.0001
**Fertilization Rate**	1,04	5,56	1,04	1,72	p<0.0001
**AMH (ng/mL)**	1.0–3.5	1.0–3.5	<1.0	<1.0	-
**AFC**	7–12	7–12	<5–7	<5–7	-
